# Fiber-Rich Cranberry Pomace as Food Ingredient with Functional Activity for Yogurt Production

**DOI:** 10.3390/foods11050758

**Published:** 2022-03-06

**Authors:** Laurita Varnaitė, Milda Keršienė, Aušra Šipailienė, Rita Kazernavičiūtė, Petras Rimantas Venskutonis, Daiva Leskauskaitė

**Affiliations:** Department of Food Science and Technology, Kaunas University of Technology (KTU), Radvilėnų Street. 19, 50254 Kaunas, Lithuania; laurita.varnaite@ktu.lt (L.V.); milda.kersiene@ktu.lt (M.K.); ausra.sipailiene@ktu.lt (A.Š.); rita.kazernaviciute@ktu.lt (R.K.); rimas.vesnkutonis@ktu.lt (P.R.V.)

**Keywords:** dietary fiber, cranberry, yogurt, rheological properties, digestibility

## Abstract

In this study, different amounts (from 2% to 4.5%) of dietary fiber-rich cranberry pomace (CP) were added to yogurt before or after fermentation to increase dietary fiber content without changing the textural properties of the product. The addition of CP reduced whey loss, improved the firmness and viscosity, increased the total phenol compound content and the antioxidant capacity values (DPPH•, ABTS, and ORAC) of the yogurt in a dose-dependent manner, and had no significant effect on the viability of the yogurt culture bacteria. For all CP-supplemented yogurt samples, the bioaccessibility index of the polyphenols after in vitro intestinal phase digestion was approximately 90%. However, yogurt with CP added before fermentation exhibited a significantly (*p* < 0.05) lower degree of protein hydrolysis post-gastric and post-intestinal than the yogurt with CP added after fermentation. Yogurt supplemented with 4.5% CP could be considered a good antioxidant dairy product and a good source of dietary fiber.

## 1. Introduction

Cranberries (*Vaccinium macrocarpon* L.) are highly valued as a source of polyphenols, carotenoids, vitamins, minerals, organic acids, and fiber [1,2]. These berries are commonly consumed fresh or used to produce juice, concentrates, and jam. After processing, about 20% of the fruit remains as pomace or press cake [3]. Recently, some attempts have been made to use this by-product, such as polyphenol extraction [4], feed supplementation in poultry [5], or a natural functional ingredient in dried fermented sausages [6]. However, there have been no attempts to apply cranberry pomace (CP) as a dietary fiber-rich functional ingredient to yogurt production to the authors’ knowledge.

The chemical composition of CP reported in the literature reveals a high amount of dietary fiber. In the dry weight of pomace, the fiber content ranges from 58.7% to 71.2% [3,7]. CP contains a variety of dietary fiber in compounds such as cellulose, lignin, pectin, and hemicelluloses [8]. The association of the solubility of dietary fibers and health-related benefits has previously been recognized. Soluble dietary fiber can effectively improve glycemic control and insulin sensitivity in patients with Type 2 diabetes [9] and act as a source of prebiotic oligosaccharides upon partial hydrolysis [10]. Insoluble fiber, which is porous and poorly fermented, can improve gastrointestinal function via gut microbial ecology and key metabolites originating from gut microbiota, such as short-chain fatty acids [11]. CP also has antioxidant potential from different phenols, making it a valuable ingredient. Previous studies have shown considerable interest in using CP as a phenol-rich functional ingredient against foodborne pathogens [6,12].

Former studies on the utilization of CP in food have assessed its potential effects on consumer health. Little attention was paid to the technological properties of CP and their possible effect on the quality of the food products. These pomaces were shown to have good swelling, water-holding capacity, and gelling-texturizing capability and have been successfully used to improve the structure of food products.

We presume that the addition of CP to the formulation of the yogurt will yield a high value-added product able to decrease fat content without changing the textural properties of the product, increase the intake of valuable dietary fibers and polyphenols, and ensure the high stability of the acidic dairy product during storage. However, the presence of polyphenols with antimicrobial properties and the high acidity of CP makes it difficult to secure the viability of the yogurt bacteria and ensure good textural properties. The bioaccessibility of the nutrients (e.g., proteins and polyphenols) in yogurt enriched with dietary fiber-rich CP should also be considered.

This study aimed to evaluate the effect of adding dietary fiber-rich CP on the physicochemical, rheological, and antioxidant properties and the bacterial viability of yogurt. Different amounts of CP (0%, 2%, 3%, and 4.5%) were added to the yogurt formulations before or after fermentation. The CP-supplemented yogurt was investigated using simulated gastrointestinal digestion, and the digestibility of proteins and release of phenols were determined during different stages of digestion.

## 2. Materials and Methods

### 2.1. Materials

Fiber-rich CP was made from frozen cranberries donated by the Fudo (Kaunas, Lithuania). The berries were thawed and pressed in a Philips HR1880/01 juicer. The resulting pomace was immediately dried in a hot air dryer at 35 °C (final moisture content 5.83%), ground in a high-speed centrifugal mill (Retsch ZM200, Haan, Germany) using a 0.5 mm sieve. The resulting CP powders were stored in sealed glass pots and refrigerated at 4 °C. Before yogurt preparation, the CP powder was pasteurized at 75 °C for 30 min in a temperature-controlled incubator (Termaks, Norway).

Skimmed milk powder containing 35.5 g/100 g of protein, 51.0 g/100 g of lactose, and 4.0 g/100 g of water was purchased from Marijampoles konservai, Lithuania. Yogurt culture, containing a mixture of *Streptococcus thermophilus* and *Lactobacillus bulgaricus* (Yo-Flex^®^, Chr. Hansen, Hoersholm, Denmark) was donated by Baltvita (Kaunas, Lithuania).

For in vitro digestion analysis, analytical grade chemicals were supplied by Sigma-Aldrich Chemie GmbH (Steinheim, UK).

### 2.2. Production of Yogurt Fortified with Cranberry Pomace

Skimmed milk powder was mixed with distilled water (16 g/100 mL) and left at room temperature (20 °C) for 2 h to hydrate. The mixture was poured into sterilized 250-mL glass beakers and heat-treated at 80 °C for 30 min in a water bath (Thermolab, GFL 1092, Potsdam, Germany). Then it was cooled in an ice bath to 40 °C. The starter culture (5 mL/100 mL) was inoculated into pasteurized milk, gently mixed, and incubated for 4 h at 42 ± 0.5 °C in an incubator (Termaks, Norway) until the pH reached 4.6 ± 0.05. After fermentation, the yogurt samples were cooled to 10 °C and stored at 4 °C for 24 h. In the case of CP addition after fermentation, 2%, 3%, and 4.5% CP were added to yogurt; in the case of CP addition before fermentation, 2%, 3%, and 4.5% CP were added to the pasteurized milk. Samples were stored at 4 °C for 7 days, and analyses were carried out.

### 2.3. Methods for Characterization of Yogurt with Cranberry Pomace

#### 2.3.1. Chemical Composition of Cranberry Pomace and Yogurt with Cranberry Pomace

The protein content was analyzed according to the Kjeldahl procedure (conversion factors of nitrogen to crude protein—5.3 for CP and 6.38). The fat content was determined by acid hydrolysis and subsequent extraction using petroleum ether in a Soxhlet apparatus. The soluble dietary fiber (SDF) and insoluble dietary fiber (IDF) were determined according to the enzymatic-gravimetric method using a total dietary fiber kit (Megazyme, Ireland) based on AOAC 991.43.

#### 2.3.2. Syneresis

Syneresis was determined by centrifuging 10× *g* of yogurt sample at 1600 rpm for 10 min using a centrifuge (MPW Med. Instruments MPW-260R, Warszawa, Poland). After centrifugation, the supernatant was drained off and weighed on a laboratory analytical balance. The syneresis was expressed according to the following equation:

(1) $$\mathrm{Syneresis}\;(\%)=\frac{\mathrm{weight}\;\mathrm{of}\;\mathrm{supernatant}}{\mathrm{weight}\;\mathrm{of}\;\mathrm{sample}}\times 100$$

#### 2.3.3. Particle Size and Distribution

The droplet size distribution of the yogurt samples was determined by laser diffraction with a Mastersizer 2000 (Malvern Instruments, Worcestershire, UK). A sample was added to circulating distilled water (1400 rpm) to give the 10%–15% obscuration rate. The average droplet size was expressed as the volume-weighted average diameter (D_4,3_).

#### 2.3.4. Rheology Assessment

The rheological characteristics of the yogurt samples were measured using a Physica MCR101 rheometer (Anton Paar GmbH, Graz, Austria) equipped with a concentric cylinder system consisting of a cup (d_inner_ = 27 mm; d_outer_ = 35 mm) and a bob with a recess at the bottom (d = 24.003 mm, h = 24.938 mm). The flow curves were obtained by measuring the shear stress as a function of the shear rates from 0 to 500 s^−1^ (up and down sweeps) at 20 °C. The flow behavior was described by the Bingham model, expressed according to the following equation:

τ = τ_o_ + Kγ (2)

where τ represents the shear stress (Pa); τ_o_ is the yield stress; K is a consistency factor (Pa s^n^), and γ; is the shear rate (s^−1^).

A mechanical spectra assay was performed using the same equipment described previously, and a frequency sweep was performed for each sample with a frequency range between 0.01 and 100 Hz at a constant strain of 0.5%. The rheological parameters determined were the elastic modulus (G′), the viscous modulus (G″), and the loss tangent (tan δ = G″/G′).

#### 2.3.5. Scanning Electron Microscopy

Scanning electron microscopy (SEM) was performed using an FEI Quanta 200 FEG scanning electron microscope (Eindhoven, The Netherlands). Yogurt samples were freeze-dried (Zirbus Technology, Bad Grunt, Germany) for 48 h (the temperature in the condenser of the freeze-drying unit was −45 °C). Lyophilized yogurt powders were placed on the SEM stubs using double-sided adhesive carbon tape. The samples were analyzed in low vacuum mode at an accelerated voltage of 10 kV.

#### 2.3.6. Lactic Acid Bacteria (LAB) Viable Cell Counts

Viable lactic acid bacteria (LAB) cell counts in yogurt were determined using the plate counting method. A sample of 1 g was mixed with 9 mL of sterile physiological saline (0.85 % wt/vol NaCl), and a series of dilutions were plated for counting the LAB on De Man, Rogosa, and Sharpe (MRS) agar (Biolife, Italia). Incubation was under aerobic conditions using a temperature-controlled incubator (Termaks, Norway) at 42 ± 0.5 °C for 48 h. LAB colonies were counted and expressed as the colony-forming unit (CFU) per gram.

#### 2.3.7. Total Phenol Content

Ten g of the yogurt sample was weighed into a 50-mL Falcon tube, mixed with 20 mL of acidified methanol (methanol containing 100 μL conc. HCl), and incubated overnight at 4 °C. The following day, the mixtures were centrifuged at 3000 rpm for 10 min at 4 °C using an MPW-260/R/RH centrifuge (MPW MED. Instruments, Warsaw, Poland). After centrifugation, the supernatant was filtered through filter paper into a 50-mL flask and concentrated by a rotary evaporator (RV 10 D S40, IKA, Staufen, Germany) at 32 °C. The concentrated solution was restored with 15 mL of methanol and stored at −18 °C until the total phenol content and antioxidant activity were determined.

The total phenol content in yogurt was determined with the Folin–Ciocalteu reagent according to the method of Slinkard and Singleton (1977) [13]. The results were expressed in mg of gallic acid equivalents per g of yogurt (µg GAE/g sample).

#### 2.3.8. Antioxidant Activity

The methanol extracts prepared for total phenol content analysis were centrifuged at 14,000 rpm for 5 min using a Velocity 14 centrifuge (Dynamica GmbH, Wiener Bundesstrasse 23, AT-5300, Salzburg-Mayrwies, Austria) and then DPPH radical scavenging capacity, ABTS radical scavenging activity and ORAC analysis were performed by the following methodologies.

##### DPPH Radical Scavenging Capacity

The DPPH radical scavenging capacity of the methanol extracts against stable DPPH• was determined by a slightly modified spectrophotometric method using a Fluostar Omega (BMG LABTECH, Ortenberg, Germany) microplate reader. The antioxidant capacity of each sample is expressed as μM in Trolox equivalents (TE)/g DW.

##### ABTS Radical Scavenging Activity

An ABTS•+ test was performed according to the protocol by Re et al. (1999) [14]. A 3-μL aliquot of sample was mixed with 300 μL ABTS•+ solution, and the absorbance was measured at 734 nm using a Fluostar Omega microplate reader against a PBS blank. The final radical scavenging values were calculated using a regression equation (y = 99.766x + 2.4483, R^2^ = 0.99) of the Trolox concentration. The antioxidant capacity of each sample was expressed as μM in TE/g DW.

##### ORAC Analysis

The ORAC analysis was performed as described by Dávalos et al. (2004) [15], Ganske (2006) [16], and Prior et al. (2003) [17] by using fluorescein as a fluorescent probe. The reaction was carried out in 75 mM phosphate buffer (pH 7.4), and a stock solution of fluorescein was prepared according to Prior et al. (2003) [17]. A mixture of 25 μL of the methanol extract and 150 μL of fluorescein (of PBS to obtain the fluorescein solution of 95.68 nmol/L for the subsequent assays) was prepared and placed in a transparent microplate and preincubated for 15 min at 37 °C, followed by the rapid addition of AAPH solution (25 μL; 240 mM). The microplate was immediately placed in the Fluorstar Omega microplate reader, automatically shaken prior to each reading, and the fluorescence was recorded every cycle (1 min × 1.1), for a total of 120 cycles. The 485 nm excitation and 520 nm emission filters were used.

Raw data were exported from the Mars software for further calculations in Excel 2003 (Microsoft, Redmond, WA, USA). The antioxidant curves (fluorescence versus time) were first normalized, and the area under the fluorescence decay curve (AUC) was calculated from the normalized curves according to the following equation:

(3) $$\mathrm{AUC}=1+\frac{f_{i}}{f_{0}},$$

where *f*_0_ is the initial fluorescence reading at cycle 0, and *f_i_* is the fluorescence reading at cycle *i* (cycle time in minutes).

The final ORAC_FL_ values were calculated using a regression equation (y = 0.1006x + 8.2753, R^2^ = 0.99) between the Trolox concentration and the net area under the fluorescein decay curve. The PBS solutions of Trolox with known concentrations ranging from 5 to 250 µM/L were used for calibration. The antioxidant capacity of each sample is expressed as μM in TE/g DW.

### 2.4. Methods for Characterization of the Digestibility of Yogurt with Cranberry Pomace

#### 2.4.1. In Vitro Digestion Model

In vitro digestion of the yogurt was performed according to the protocol described by Minekus et al. (2014) [18]. This model comprises three parts that simulate the digestion environment: the mouth, stomach, and intestine. Briefly, yogurt samples (5 g) and glass beads (2 g) were incubated and sequentially mixed for 2 min with 5 mL of simulated saliva fluids, 120 min with 10 mL of simulated gastric fluid containing pepsin (2000 U/mL of digest) (post gastric sample) and 120 min with 20 mL of intestinal fluid containing pancreatin (100 U/mL of digest), lipase (2000 U/mL of digest), and bile salts (10 mM of digest) (post intestinal sample). The pH of the stomach phase was adjusted to 3 with 6M HCl, and the pH of the intestinal phase was adjusted to 7 with 6M NaOH. During the whole process, samples were swirled at 140 rpm in a shaking water bath (Thermolab, GFL 1092, Potsdam, Germany) at 37 °C to simulate the motility and temperature of the gastrointestinal tract. The digestion process of the samples was stopped by cooling the samples to 0 °C–4 °C in ice water. After cooling, the samples were centrifuged at 4000 rpm at 4 °C (MPW Med. Instruments MPW-260R) and filtered. For each time point sampled (0 min and 120 min of the gastric (G) and intestinal (D) phases), aliquots were taken to analyze the free amino terminals and the total phenol content. These were stored in Eppendorf tubes at −18 °C until the analysis was performed.

#### 2.4.2. Free α-Amino Groups by Fluorescamine Assay

The level of free amino terminals in digested yogurt was determined according to the protocol described by Jansson et al. (2014) [19]. Quantification was achieved by calculating leucine equivalents using an external leucine standard curve.

The degree of proteolysis was calculated using the following equation:

(4) $$DH_{protein}\left(\%\right)=\frac{h}{h_{tot}}\times 100$$

where *h* is the amount of N-terminal amines at each time point of in vitro digestion and *h_tot_* is the total amount of N-terminal amines determined after full hydrolysis with HCl.

#### 2.4.3. The Bioaccessibility Index (BI) of the Phenol Compounds

The BI of the phenol compounds was calculated as the ratio of the total phenols in the digested yogurt using the following equation:

(5) $$\mathrm{BI}\;(\%)=\frac{TPC_{1}}{TPC_{2}}\times 100$$

where *TPC*_1_ is the total gastric (or intestinal) phenol content at the initial digestion phase (0 min) and *TPC*_2_ is the total gastric (or intestinal) phenol content at the final digestion phase (120 min).

### 2.5. Statistical Analysis

All tests were performed in triplicate. The results are expressed as calculated means and standard deviations. Statistical analysis of the data was carried out by paired *t*-test. Statistical analysis was carried out with an analysis of variance (ANOVA) at *p* < 0.05.

## 3. Results and Discussions

### 3.1. Physicochemical Characterization of Yogurt Fortified with Fiber-Rich Cranberry Pomace

#### 3.1.1. Chemical Composition of Cranberry Pomace and Yogurt Supplemented with Cranberry Pomace

The chemical composition of the CP is presented in Table 1. The fat, protein, soluble fiber, and insoluble fiber content of the CP were 7.13 ± 0.39, 7.6 ± 0.09, 12.74 ± 0.09, and 59.93 ± 1.46 g/100 g, respectively. Data taken from the literature revealed significant differences between the compositions of berry-based sources of fiber. The dietary fiber content in the pomace and the ratio between the soluble and insoluble fiber depend on the source and processing conditions during fiber isolation [20]. Our results regarding insoluble fiber content are compatible with Reißner et al. (2019) [21], who analyzed the chemical composition of dried blackcurrant, redcurrant, gooseberry, rowanberry, and chokeberry pomace. They reported that the insoluble fiber content in dried pomace varied between approximately 50.0 and 60.0 g/100 g DM and that the soluble fiber content varied between approximately 4.0–7.0 g/100 g DM. We found a higher amount of soluble fiber in CP than that reported for the other berry pomaces, but our results are in line with the data presented by Islam et al. (2020) [5], who reported that the pomace obtained from organic cranberries contained on average 46.3 and 15.5% (DM) of coarse and fine fiber, respectively. Consequently, CP may be considered rich in dietary fiber and used in the formulation of yogurt supplemented with dietary fiber.

The chemical composition of yogurt supplemented by different amounts of CP is also presented in Table 1. The total solids content in the yogurt ranged from 13.84% to 17.92%; the higher the added CP content was, the higher the total solids content was. The increase in added CP content caused an increase in fat content from 0.20% to 0.44% and protein content from 5.83% to 6.01%. As for the dietary fiber content, the addition of 2% CP increased dietary fiber content by approximately 1.45%, while the addition of 4.5% CP increased the content of dietary fiber by approximately 3.27% compared with plain yogurt. Therefore, yogurt made with 4.5% CP could be claimed as “a source of fiber” or “containing fiber”. This yogurt formulation fulfills the European Food Safety Authority (EFSA) requirement for nutrient claims of having at least 3 g/100 g of dietary fiber [22].

#### 3.1.2. Rheological and Structural Properties of Yogurt Fortified with Fiber-Rich Cranberry Pomace

**Figure 1:**
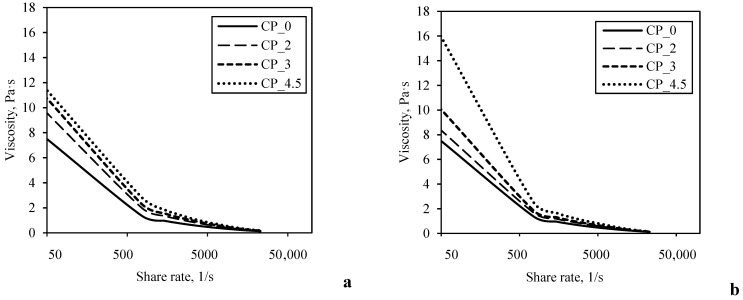
Viscosity curves of yogurt made by adding different amount of CP after (**a**) and before fermentation (**b**).

The viscosity curves (Figure 1) revealed the pseudoplastic shear thinning behavior of plain yogurt (CP_0) and those made with CP (i.e., CP_2, CP_3, CP_4.5). The viscosity of all of the samples decreased continuously with increasing shear rate throughout the entire shear rate range studied. However, when the CP was added to the yogurt, different effects were observed on the rheological characteristics depending on the stage during which the CP addition occurred. The rheological characteristics estimated from the rheological measurements are presented in Table 2. The addition of 2.0%–4.5% CP after the fermentation of the yogurt moderately increased the yield stress (τ_o_) and the consistency index (K) from 12.95 ± 0.3 to 24.32 ± 1.46 Pa and from 0.12 ± 0.00 to 0.22 ± 0.02 Pa s^n^, respectively. The significant (*p* < 0.05) increase of these rheological characteristics was observed only compared to the control sample. When the CP was added to the yogurt before fermentation, significantly higher values of the yield stress (τ_o_) and the consistency index (K) were recorded for the samples containing higher amounts of CP. The highest rheological characteristic values were determined when 4.5% CP was added to the yogurt before fermentation; τ_o_ was 36.16 ± 0.85 Pa and K was 0.29 ± 0.00 Pa s^n^. It could be assumed that dietary fiber-rich CP with good water-binding capacity was responsible for such rheological characteristics of yogurt enriched by CP.

Our observations agree with those reported by Wang et al. (2020) [23], who fortified yogurt with apple pomace by adding it after fermentation. In their study, the incorporation of 3% apple pomace significantly increased the viscosity of the yogurt in comparison with the control sample. Still, the opposite results have also been found. Tseng et al. (2013) [24] found that yogurt made with 3% of wine grape pomace (added after fermentation) had lower viscosity than plain yogurt. Sometimes reported rheological properties of fortified yogurt are not comparable due to the differences in pomace and yogurt preparation and the analytical methods used.

**Figure 2:**
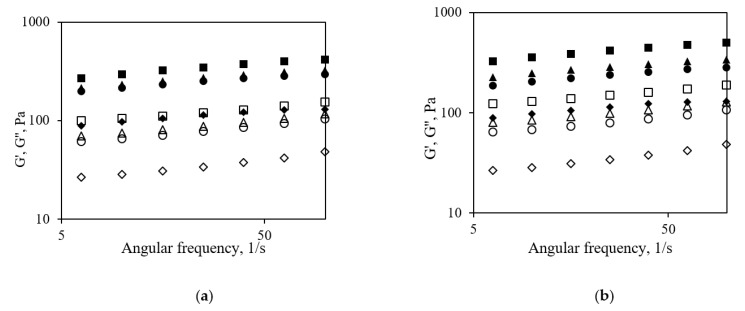
Frequency sweep tests of yogurt made by adding different amounts of CP after (**a**) and before fermentation (**b**). ♦, ◊—CP_0; ●, ○—CP_2; ▲, △—CP_3; ■, □—CP_4.5. Filled symbols represent storage modulus (G′), empty symbols represent loss modulus (G″).

The results from the frequency sweep tests demonstrated that all samples reflected characteristics typical for weak gels, as G′ > G″ throughout the entire frequency range (Figure 2). G′ and G″ curves occurred in the form of parallel straight lines, showing only very slight slopes. The G′ values at an angular frequency of 25 1/s significantly (*p* < 0.05) increased from 138 ± 18.08 to 419 ± 8.08 Pa with increased amounts of added CP, regardless of whether it was added before or after fermentation (Table 2). However, yogurts supplemented with CP after fermentation showed lower G′ values than yogurts with the same amount of CP added before fermentation. The G′ values indicate the number and strength of bonds in the casein network. Post-fermentation addition of CP may be responsible for the disruption of the network causing lower G′ values. Adding CP before fermentation causes casein network, which entraps CP more effectively, thereby creating a more compact network with higher G′ values.

The values of the loss tangent (tan δ) ranged from 0.3 for the control sample to 0.37 and 0.34 for the yogurt fortified with 4.5% CP added before and after fermentation, respectively. Gel-like structures are resistant against whey separation (syneresis) and show high stability against low agitation if tan δ is between 0.2 and 0.3. This is confirmed by the results of syneresis (Table 2). The highest syneresis (24 ± 2%) was registered for the control sample. Incorporating CP into the yogurt structure significantly (*p* < 0.05) reduced the amount of whey separation. The CP-fortified yogurt with 4.5% CP added before fermentation held more whey than the CP-fortified yogurt with the same amount of CP added after fermentation. Syneresis occurs due to the inability of the yogurt gel to retain the serum phase because of weakening of the casein network and leads to the release of whey [25]. The increase in total solids content positively influences the network density and reduces the syneresis of fermented milk [26]. In agreement with our results, studies of yogurt supplemented with fiber derived from oranges [27] have also documented a decrease in syneresis. According to Sah et al. (2016) [28], an incompatibility between milk proteins and polysaccharides can occur in yogurt and is affected by the amount of dietary fiber added. At low concentrations (<1%) of orange fiber, decreased viscosity and increased syneresis have been found, while higher amounts of fiber improved the textural properties and viscosity of the yogurt [29].

**Figure 3:**
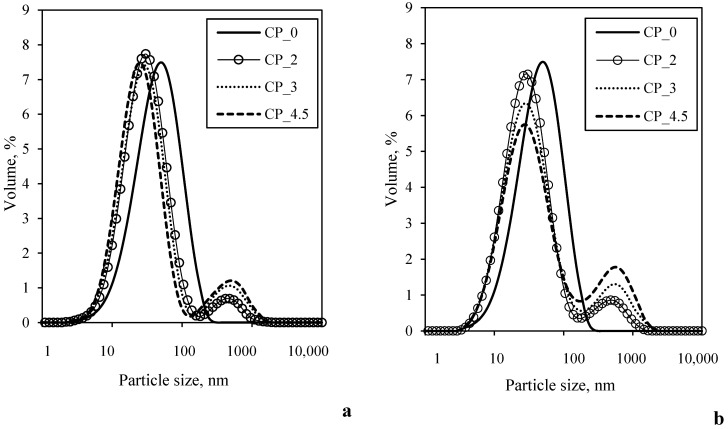
Particle size and distribution of yogurt made by adding different amount of CP after (**a**) and before fermentation (**b**).

Data from the literature reveals that the changes in yogurt structural properties were related not only to the fiber content but also to the particle size of the fiber [27]. Our data about the particle size distribution in yogurt (Figure 3) describes yogurt as a network of aggregated casein particles with larger CP particles dispersed in it. The plain yogurt samples showed a monomodal particle size distribution between ~5.0 and ~200.0 nm (Figure 3), while a bimodal particle size distribution was observed for the CP-supplemented yogurt. The shape of this bimodal curve was dependent on the timing of the CP addition. In the case of yogurt supplemented with CP after fermentation, most of the particle sizes were in the range 3.0–138.0 nm, and only a small number of them were in the range 140.0–1250.0 nm (Figure 3a). Increasing the amount of added CP caused an increase in the height of the second peak, representing CP particles, and had a very slight effect on the height of the first peak, representing aggregated casein particles. In the case of yogurt supplemented with CP before fermentation, the first peak was in the range 3.0–160.0 nm and depended on the amount of added CP; the height of the first peak decreased with increasing amounts of added CP (Figure 3b). Although the curve also had two peaks, in this case, the separation between the peaks is not as clear as in the first case. The second peak showed the distribution of particles in the interval 180.0–2200.0 nm and was also dependent on the amount of added CP. It can be assumed that adding CP to the system before the casein network formed invoked the complexation between milk proteins and polysaccharides from CP, which drove the more intensive formation of larger particles in the structure of yogurt.

**Figure 4:**
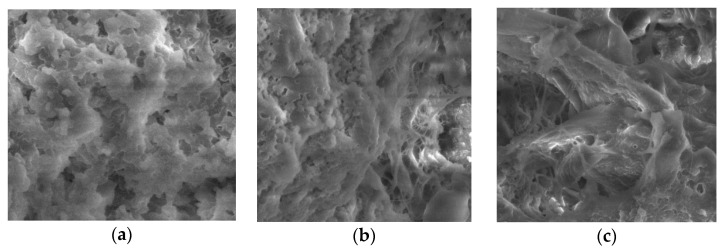
SEM micrographs of yogurt without (**a**) and with 4.5% of CP added after (**b**) and before (**c**) fermentation.

Micrographs of the yogurts also showed the differences in the CP-fortified yogurt structure, particularly for high CP content. A typical casein aggregate-based network with open spaces was recorded for the plain yogurt (Figure 4a). The microstructure of the CP-supplemented yogurt was different from that of plain yogurt and depended on the timing of the CP addition. The yogurt with CP added after fermentation (Figure 4b) consisted of a three-dimensional network of casein aggregates with larger pores and heterogeneously sized channels with the CP fiber located within these void spaces. The yogurt with CP added before fermentation demonstrated two heterogeneously blended networks: a rough appearance representing CP fiber and another more compact protein network, which seemed to be embedded into the first one (Figure 4c). 

Amirtha et al. (2014) [30] studied the microstructure, rheological, and textural properties of yogurt gels with 1 or 2% carrot fiber added before fermentation, concluding that: (a) the carrot fiber was individually embedded in the casein micelle network providing a “filler” effect and synergistically contributed to the rheological properties of the yogurt gel; (b) at higher concentrations the carrot fiber can also form a network, thereby hindering the formation of a connected colloidal network by the casein micelles. Kieserling et al. (2019) [27] pointed out that fiber may act as an active and inactive filler in protein gels depending on the composition, that is, the insoluble and soluble fiber content. According to our results, CP has a high soluble fiber content (12.74% ± 0.09%). Gouw et al. (2017) [3] showed that CP is high in pectin; they found 10.58% pectin in the dry matter of CP. The adsorption and interconnection of the casein micelles with pectin can occur at low concentrations of the latter [31]. Additionally, pectin can make complexes with calcium released from casein micelles [32]. Both possible interactions suggest CP acts as an active filler with additional stabilization of the serum phase in the well-developed casein network. Moreover, as CP contains 59.93% ± 1.46% of insoluble dietary fiber (cellulose, lignin, etc.), hydration and swelling of fiber molecules distributed in the serum phase of the casein network can contribute significantly to the structural properties of the yogurt by water immobilization and the formation of a connected colloidal network. The stage of CP addition seems to be of great importance in these processes.

#### 3.1.3. Viability of Yogurt Culture Bacteria in the Yogurt Fortified with Fiber-Rich Cranberry Pomace

**Figure 5:**
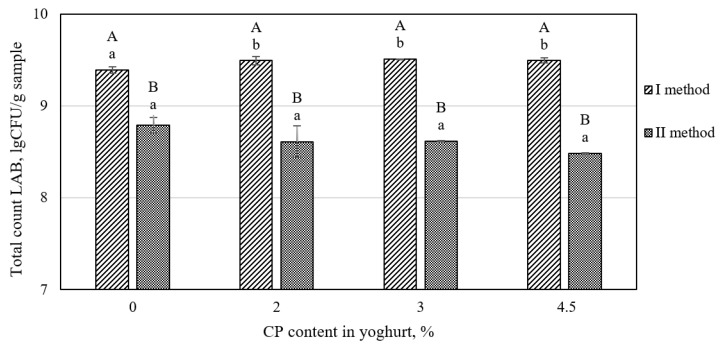
Viability of lactic acid bacteria (LAB) in yogurt made by adding different amounts of CP after (I method) and before fermentation (II method). Values are reported as means ± standard deviation; lower case letters indicate significant (*p* < 0.05) differences between different CP content in yogurt; upper case letters indicate significant (*p* < 0.05) differences between different CP addition methods.

An appropriate concentration of viable LAB is especially important in the yogurt fortified by CP due to claims about the antimicrobial properties of cranberries [33]. As shown in Figure 5, adding CP after fermentation slightly increased the number of LAB cultures compared with the control yogurt (without CP). When CP was added before fermentation, a modest decrease in LAB viability was detected compared to the control yogurt. The increased amounts of added CP had no significant effect on the LAB viability in both cases. It is very important that yogurt fortified with CP has a higher concentration of viable LAB than that required by the Codex Alimentarius (10^7^ CFU/g) [34]. Previously, supplementation of total dietary fiber from apple, banana, or passion fruit processing by-products has been reported as resulting in no changes in the levels of *S. thermophilus* and *L. bulgaricus* starter culture counts in yogurt [35]. However, the incorporation of CP into fermented dry sausages affected the starter culture population, and the growth of lactic acid bacteria was stimulated in the presence of 1.7 and 2.25% CP [6].

#### 3.1.4. Antioxidant Activity of Yogurt Fortified with Fiber-Rich Cranberry Pomace

The DPPH, ABTS, and ORAC antioxidant activity demonstrated significant variability (*p* < 0.05) among yogurts with and without added CP (Table 3). Increased amounts of CP significantly (*p* < 0.05) increased the antioxidant activity of the fortified yogurt compared to the control yogurt. These findings agree with those of Sah et al. (2016) [28] and Ribeiro et al. (2021) [36], who reported increased antioxidant activity in yogurt produced with pineapple waste powder and olive pomace powder, respectively. Our study recorded the highest DPPH/ABTS/ORAC activity for the samples with 4.5% CP. The increase in the antioxidant activity of CP-fortified yogurt may be explained by the addition of cranberry polyphenols, which are strong antioxidants [4]. All yogurt samples fortified with CP exhibited a significant (*p* < 0.05) increase in total phenol content from 62.67 to 167.39–188.95 µg GAE/g sample with the increase of added CP from 2% to 4.5%. Similar results were reported for the apple pomace- and black mulberry puree-fortified yogurts [23,37]. Furthermore, CP is a good source of pectin [8]. According to the findings of Sabater et al. (2020) [38], the galacturonic acid in pectin also contributes to the antioxidant activity of yogurt. It should also be noted that Folin–Ciocalteu’s reaction is based on a single electron transfer; therefore, in the case of the control samples, the TPC value most likely does not indicate the presence or absence of phenols. However, adding CP enriches the product with plant phenol antioxidants.

According to the data presented in Table 3, in yogurt, the metabolites of LAB fermentation, such as organic acids, low molecular weight peptides, glutathione, and folate, contribute to the antioxidant potential of CP-fortified yogurt along with the phenol compounds and organic acids of CP [38]. In general, all antioxidant capacity values increased with CP in a dose-dependent manner, independent of the stage at which the CP was added. However, the ABTS values were slightly higher when 3% and 4.5% CP were added before fermentation (method II), while the ORAC values were remarkably higher when the CP was added after fermentation at all concentrations (method I). This response may be explained by two main factors: (i) the differences in the chemistry behind these assays [39] and (ii) the changes in the food system during fermentation. The ABTS radical cation decolorization reaction is based on a single electron transfer, while the ORAC assay is based on the neutralization of reactive oxygen species. Therefore, the latter is better suited to the oxidation–reduction processes in biological systems. For both methods, the effect of adding 1% CP was almost similar in terms of increasing ORAC values by 0.93 and 1.05 mM TE/g. It may be assumed that fermentation products have more pronounced effects on the ABTS radical cation decolorization reaction, particularly at higher CP doses. The results obtained prove that for complex foods, particularly those containing proteins and reducing sugars, more than one method is required to demonstrate the effects of the added plant-origin antioxidants. For instance, Huang et al. (2005) [39] recommended using three assays for this purpose, including Folin–Ciocalteu, ORAC, and one electron/hydrogen atom transfer-based method.

The changes of the food system during fermentation should also be considered. Multiple studies have shown that the interactions between phenols and milk proteins mainly result from H-bonding and hydrophobic interactions [40]. Xiong et al. (2020) [41] proposed that phenols from berry pomace can be protected from degradation after complexation with protein. More intensive interaction of phenols with protein can take place during the fermentation of milk with the CP at 43 °C. After fermentation, these phenols may be released gradually, thus increasing the antioxidant activity of the CP-fortified yogurt.

### 3.2. Digestibility of Yogurt Fortified with Fiber-Rich Cranberry Pomace

In vitro digestibility analysis was conducted with yogurts containing different amounts of CP added before or after fermentation. The results are presented in Table 4.

In the CP-fortified yogurt, after the gastric stage of digestion, the BI of the phenol compounds increased with CP in a dose-dependent manner for CP added both before and after fermentation. The same tendency was observed after the intestinal phase of digestion. The BI after the intestinal stage of digestion of the CP-fortified yogurt was significantly higher (*p* < 0.05) than that determined for the CP-fortified yogurt after the gastric stage of digestion.

In contrast, the degree of protein hydrolysis during gastrointestinal digestion seemed to be affected by both the amount of CP and the timing of its addition. The degree of protein hydrolysis of all yogurt samples exhibited an increasing trend with increasing amounts of added CP. In all samples, proteins were significantly (*p* < 0.05) less degraded after the gastric stage of digestion than after the intestinal stage. In contrast, the stage during which the CP was added to the yogurt seems to be of great importance for the digestibility of proteins. The yogurt with CP added before fermentation exhibited significantly (*p* < 0.05) lower post-gastric and post-intestinal degrees of protein hydrolysis compared to the yogurt with CP added after fermentation. This result could be related to the peculiarities of the structures determined for the differently prepared yogurts. A more compact network of casein aggregates embedded into the network of CP fibers (method II) hinders the access of gastric enzymes to target residues, resulting in practically undigested proteins (post-gastric degree of protein hydrolysis: 7.95%–10.05%). As a result, proteins remaining in the stomach can greatly affect the consistency characteristics of the gastric environment [42]. According to van Aken et al. (2011) [43], this period of delayed stomach emptying can lead to feelings of gastric distension and fullness. It is also related to the slower hydrolysis of proteins by pancreatic proteases, which require more time for degradation and are more distally absorbed in the intestine [44]. According to our results, adding CP to the yogurt before fermentation will potentially improve the satiating ability of the product. In contrast, samples of the yogurt with CP added after fermentation underwent gradual and more extended digestion during the gastric and intestinal stages. The open structure of casein aggregates with large pores, heterogeneously sized channels, and CP fiber within these void spaces allows enzymes greater access to potential cleavage sites, increasing the degree of protein hydrolysis. In this case, slow emptying of the stomach can be predicted. Afterward, protein degradation products are hydrolyzed by pancreatic proteases and evenly absorbed in the upper part of the small intestine [45]. Our results are in accordance with the results of studies by Borreani et al. (2016) [45] and Morell et al. (2017) [42], which showed that the structural and textural properties of milk systems were of great importance for the digestibility of milk proteins.

## 4. Conclusions

Cranberry pomace (CP) can be used in yogurt production as a source of dietary fiber and antioxidants with strong effects on the textural and structural properties. The amount of CP and the stage during which it is added (before or after fermentation) significantly affected the rheological and structural characteristics of the yogurt. The addition of 2.0%–4.5% CP after fermentation moderately increased the yield stress (τ_o_) and the consistency index (K); however, significant (*p* < 0.05) increases in these rheological characteristics were observed only compared with the control sample. When CP was added before fermentation, significantly higher values of τ_o_ and K were recorded for the samples containing a higher amount of CP. Additionally, yogurts supplemented with CP after fermentation showed lower G′ values than yogurts with the same amount of CP added before fermentation. Microstructure analysis revealed a network of casein aggregates with the CP fiber located within the large void spaces for the yogurt with CP added after fermentation. In contrast, two heterogeneously blended networks of casein aggregates and CP fiber were observed in the yogurt with CP added before fermentation.

The added CP increased the total phenol compounds content and the antioxidant capacity (DPPH•, ABTS, and ORAC) values of yogurt in a dose-dependent manner. However, the ABTS values were slightly higher when 3% and 4.5% CP were added before fermentation (method II), while the ORAC values were remarkably higher when the CP was added after fermentation at all concentrations (method I). This behavior may be explained by two main factors: the differences in the chemistry behind these assays and the changes in the food system during fermentation. It is very important that yogurt fortified with CP had a higher concentration of viable LAB than that required by the Codex Alimentarius (10^7^ CFU/g). The increased amounts of added CP (before or after fermentation) had no significant effect on the LAB viability.

After in vitro digestion of CP-supplemented yogurt, the BI of polyphenols after the intestinal phase was approximately 90%. However, yogurt with CP added before fermentation exhibited significantly (*p* < 0.05) lower post-gastric and post-intestinal degrees of protein hydrolysis than yogurt with CP added after fermentation.

This study highlights the usefulness of cranberry pomace for the production of yogurt rich in dietary fiber. The addition of pomace to yogurt before or after fermentation has no significant effect on the textural properties or viability of LAB in the product. However, differences in the digestibility of proteins suggest that the way CP is added to the yogurt may affect the satiating ability of these products.

## Figures and Tables

**Table 1:** Chemical composition (g/100 g) of cranberry pomace (CP) and yogurt made with different amounts of CP.

Sample	Fat	Protein	Fiber	Soluble Fiber	Insoluble Fiber	Total Solids
CP	7.13 ± 0.39	7.6 ± 0.09	72.67 ± 1.55	12.74 ± 0.09	59.93 ± 1.46	96.71 ± 0.96
Yogurt with CP						
CP_0	0.00	5.68	0.00	0.00	0.00	13.84
CP_2	0.20	5.83	1.45	0.25	1.20	15.66
CP_3	0.29	5.90	2.18	0.38	1.80	16.56
CP_4.5	0.44	6.01	3.27	0.57	2.70	17.92

**Table 2:** Physicochemical and rheological properties of yogurt made by adding different amounts of CP after (I method) and before fermentation (II method).

CP Content in Yogurt, %	Rheological Characteristics from Flow Curves and Mechanical Spectra ^3^				Syneresis, %	Average Particle Size, D 4.3 (nm)
	τ_0_^1^, Pa	K ^1^ (Pa s^n^)	G′ ^2^, Pa	Tanδ ^2^		
0	12.95 ± 0.30 a	0.12 ± 0.00 a	138 ± 18.08 a	0.30 ± 0.00 a	24 ± 2 a	59.14 ± 0.86 a
	I method					
2	19.53± 1.48 bA	0.17 ± 0.00 bA	225 ± 11.72 bcA	0.31 ± 0.00 aA	13 ± 3 bA	58.35 ± 4.68 abA
3	20.90 ± 3.07 bcA	0.20 ± 0.00 cdA	261 ± 5.29 cA	0.33 ± 0.01 bA	11 ± 2 cdA	72.94 ± 3.80 bA
4.5	24.32 ± 1.46 cA	0.22 ± 0.02 dA	348 ± 19.01 dA	0.34 ± 0.00 bA	9 ± 1 dA	101.02 ± 10.83 cA
	II method					
2	15.30 ± 0.10 bB	0.17 ± 0.02 bA	233 ± 17.39 bA	0.33 ± 0.00 bB	12 ± 2 bA	67.94 ± 2.69 bA
3	18.96 ± 0.96 cA	0.18 ± 0.02 bcA	283 ± 17.44 cdA	0.34 ± 0.00 bcA	7 ± 3 cB	77.42 ± 2.94 cA
4.5	36.16 ± 0.85 dB	0.29 ± 0.00 cB	419 ± 8.08 dB	0.37 ± 0.01 cB	6 ± 1 cB	113.27 ± 7.47 dB

^1^—yield stress (τ_0_) and consistency index (K) calculated using Bingham model; ^2^—storage modulus (G′) and loss tangent (tanδ) at an angular frequency of 25 1/s; ^3^—values are reported as means ± standard deviation; lower case letters indicate significant (*p* < 0.05) differences between different CP content in yogurt; upper case letters indicate significant (*p* < 0.05) differences between different CP addition method.

**Table 3:** Total phenols content and antioxidant capacity of yogurt made by adding different amounts of CP after (I method) and before fermentation (II method).

CP Content in Yogurt, %	Total Phenols Content, µg GAE/g Sample	Antioxidant Activity		
		DPPH, μM TE/g DW	ABTS, μM TE/g DW	ORAC, μM TE/g DW
0	62.67 ± 2.73 aA	1.74 ± 0.20 aA	10.03 ± 1.12 aA	16.67 ± 1.25 aA
	**I method**			
2	125.20 ± 2.84 bA	3.44 ± 0.90 bA	19.13 ± 2.89 bA	46.39 ± 2.30 bA
3	138.40 ± 2.57 cA	3.98 ± 0.51 bA	22.44 ± 1.02 cA	47.01 ± 1.41 bA
4.5	188.95 ± 1.84 dA	3.46 ± 0.47 bA	20.96 ± 1.89 cA	53.54 ± 0.04 cA
	**II method**			
2	118.59 ± 0.14 bB	3.57 ± 0.72 bA	20.16 ± 0.72 bA	24.78 ± 0.99 bB
3	139.09 ± 3.39 cA	3.74 ± 0.17 bA	24.37 ± 1.36 cB	34.36 ± 2.56 cB
4.5	167.39 ± 0.42 dB	4.01 ± 0.63 bA	28.76 ± 1.26 dB	36.02 ± 0.91 dB

Values are reported as means ± standard deviation; lower case letters indicate significant (*p* < 0.05) differences between different CP content in yogurt; upper case letters indicate significant (*p* < 0.05) differences between different CP addition methods.

**Table 4:** Digestibility characteristics of yogurt made by adding different amounts of CP after (I method) and before fermentation (II method).

Digestion Stage		I Method			II Method		
	CP_0	CP_2	CP_3	CP_4.5	CP_2	CP_3	CP_4.5
	Protein hydrolysis degree, %						
Post gastric	11.77 ± 1.50 aA	15.34 ± 1.41 aA	20.29 ± 0.73 bA	23.89 ± 3.39 bA	10.05 ± 1.73 abB	8.42 ± 5.39 abA	7.96 ± 0.05 bB
Post intestinal	60.96 ± 10.50 aA	61.27 ± 3.02 aA	78.14 ± 2.49 bcA	82.72 ± 1.99 cA	17.82 ± 2.06 aB	18.62 ± 1.66 bB	26.08 ± 4.51 bB
	BI of phenolic compounds, %						
Post gastric	-	79.35 ± 0.47 bA	78.76 ± 0.18 bA	82.14 ± 0.69 cA	71.56 ± 0.19 aB	74.05 ± 0.28 bB	76.73 ± 3.14 bA
Post intestinal	-	93.61 ± 0.65 bA	93.59 ± 2.82 baA	93.75 ± 3.00 baA	85.64 ± 1.82 aA	85.70 ± 0.33 aA	92.42 ± 0.52 aA

Values are reported as means ± standard deviation; lower case letters indicate significant (*p* < 0.05) differences between different CP content in yogurt; upper case letters indicate significant (*p* < 0.05) differences between different CP addition methods.

## Data Availability

Data is contained within the article.
